# Supplementary material for: Complexome profiling of the Chlamydomonas psb28 mutant reveals TEF5 as an early PSII assembly factor
Source: Plant Cell. 2025 Mar 17;37(6):koaf055. doi: 10.1093/plcell/koaf055 (PMC12164590; doi:10.1093/plcell/koaf055)
Supplement: koaf055_Supplementary_Data [file koaf055_supplementary_data.zip › psb28_interactive.zip!/plots/intensity/qualityPlots/peptides/_Cre03.g145507.t1.1.html]

### Cre03.g145507.t1.1

Trivial name:   
SeqCoverage [median]: 48.30  
Mass: 27.94 kDa  
  
 

| Sequence | MissedCleavage | Mass [Da] | wtIntensity | muIntensity | Score |
| --- | --- | --- | --- | --- | --- |
| AIELLQDPRPLEEK | 1 | 1649.90 | 14451.38 | 49953.50 | 121.28 |
| EVEDTLVEFLEK | 0 | 1449.72 | 1852.23 | 0.00 | 120.09 |
| GATVESDVLANVK | 0 | 1301.68 | 13901.89 | 38782.79 | 238.51 |
| ILLEEVDSSFFSSSA | 0 | 1629.78 | 5969.24 | 0.00 | 132.91 |
| RVAEVSPATEADPSIAAATR | 1 | 2011.03 | 6644.65 | 14913.61 | 129.20 |
| SALDAVR | 0 | 730.40 | 2656.27 | 5866.87 | 94.26 |
| TNIDPASTNILR | 0 | 1313.69 | 11840.31 | 32095.88 | 188.96 |
| TPFDILTFGPR | 0 | 1262.67 | 10184.77 | 3545.32 | 195.70 |
| VAEVSPATEADPSIAAATR | 0 | 1854.93 | 5131.17 | 8719.73 | 128.96 |
| VGAGALLSVPER | 0 | 1167.66 | 14597.81 | 44925.26 | 170.47 |
|  |  |  | 87229.7 | 198803.0 |  |
